# A Study on Thin Cooling Layers Between the Cooling Channel and Cavity in the Injection Molding Process for Mold Temperature Control to Enhance Weld Line Flexural Strength in Plastic Products

**DOI:** 10.3390/polym17212831

**Published:** 2025-10-23

**Authors:** Tran-Phu Nguyen, Pham Thi Mai Khanh, Pham Son Minh, Tran Minh The Uyen, Bui Chan Thanh

**Affiliations:** 1 Faculty of Vehicle and Energy Engineering, HCMC University of Technology and Education, Ho Chi Minh City 71307, Vietnam; phunt@hcmute.edu.vn; 2 Faculty of Engineering and Technology, Nong Lam University, Ho Chi Minh City 71307, Vietnam; maikhanh.pham40@gmail.com; 3 Faculty of Mechanical Engineering, HCMC University of Technology and Education, Ho Chi Minh City 71307, Vietnam; minhps@hcmute.edu.vn (P.S.M.); uyentmt@hcmute.edu.vn (T.M.T.U.)

**Keywords:** thin-walled mold, cooling layer, heat transfer, mold temperature, injection molding, weld line, Taguchi method

## Abstract

Weld lines in injection-molded plastics often act as structural weak points that reduce mechanical performance. Enhancing weld line strength is therefore essential to improve product reliability and service life. This study aims to develop and validate an injection mold system capable of localized cavity temperature control to strengthen weld line regions. A specialized mold with an integrated cooling layer was designed to enable rapid thermal response during molding. The Taguchi method was applied to optimize three key parameters—part thickness, melt temperature, and injection pressure—to maximize weld line flexural strength. Experiments based on an L25 orthogonal array revealed that weld line stress varied significantly across parameter combinations, with a maximum of 109.23 MPa. A subsequent validation test conducted under the optimal conditions (250 °C melt temperature, 1.5 mm part thickness, and 16 MPa injection pressure) achieved an enhanced weld line stress of 121.88 MPa, confirming the reliability of the Taguchi-based optimization. Among the factors studied, part thickness had the greatest influence, followed by injection pressure, while melt temperature had the smallest effect. These results demonstrate that combining cavity temperature control with systematic parameter optimization provides an effective strategy to enhance weld line strength in high-performance plastic components.

## 1. Introduction

Plastic products manufactured by injection molding play a pivotal role in a broad spectrum of industries, ranging from automotive, electronics, and consumer goods to medical devices and packaging. The widespread adoption of this manufacturing technology is largely attributable to its ability to produce high-precision components with complex geometries, excellent surface finish, and consistent dimensional stability at high production rates. Despite these advantages, the performance and durability of injection-molded parts are significantly affected by weld lines. These defects form when two or more flow fronts of molten polymer meet and solidify without complete molecular entanglement. Weld lines often act as stress concentrators, resulting in local reductions in mechanical strength, surface quality degradation, and, in some cases, premature product failure. This limitation has driven extensive research into understanding the mechanisms of weld line formation, its impact on mechanical properties, and the process modifications necessary to mitigate its adverse effects.

One of the most critical process parameters affecting weld line quality is the mold temperature at the meeting front of the melt streams. When the polymer melt encounters a relatively cool mold surface, it undergoes rapid solidification at the interface, restricting molecular diffusion across the weld line and thereby diminishing interfacial strength. Consequently, precise temperature control within the mold cavity has been recognized as a key strategy to enhance weld line strength and overall product performance. The importance of mold temperature extends beyond weld line quality; it also influences residual stress, shrinkage, and warpage, all of which determine the dimensional stability and mechanical integrity of the final product.

In this context, advanced cooling system designs—particularly conformal cooling channels and localized heating methods—have emerged as promising solutions to maintain optimal mold surface temperatures. These innovations offer the dual benefit of improving weld line strength while simultaneously controlling warpage, thus contributing to superior product quality. However, the interplay between mold temperature control, mechanical properties, and part deformation is complex. A comprehensive understanding of both the thermal and rheological behavior of the polymer during injection molding is therefore required.

Several studies have investigated the formation and characterization of weld lines in injection-molded parts. Do et al. [1] studied the effect of thermoplastic polyurethane (TPU) content on the weld line characteristics of polypropylene (PP) and acrylonitrile–butadiene–styrene (ABS) blends. They found that adding TPU changed the interfacial adhesion and the morphology near the weld line. These changes affected both the mechanical properties and the appearance of molded parts. The study shows that material composition strongly influences weld line quality. It also suggests that elastomeric modifiers can improve weld line strength in polymer blends. Minh et al. [2] investigated the weld line strength of composite parts molded with different venting systems. They reported that the venting design influenced the filling process, the removal of trapped gas, and the local pressure distribution. As a result, venting conditions had a direct impact on weld line strength. Better venting helped reduce weld line defects and improved the mechanical performance of molded parts. This study highlights the importance of mold design in controlling weld line quality. Raz and Sedlacek [3] examined the influence of melt temperature on weld line strength in polypropylene parts. They reported that strength increases with temperature up to about 210 °C, but declines at higher levels due to thermal degradation. Mold-flow analysis indicated that shear and friction can elevate the actual melt temperature by up to 18%. The study suggested an optimal range of 190–210 °C for maximizing weld line strength. In addition, Ozcelik [4] applied the Taguchi method to optimize injection parameters for polypropylene weld line strength, providing a statistical basis for process optimization. These works collectively underscore that weld line formation is highly sensitive to thermal conditions during filling and packing stages, and that mold temperature control offers a viable route to improving mechanical properties at these critical regions.

Beyond weld line defects, warpage—a form of part distortion caused by non-uniform shrinkage—remains a prevalent challenge in injection molding. Warpage is influenced by cooling rates, thermal gradients, part geometry, and material properties. Nian et al. [5] demonstrated that localized mold temperature control could be used to minimize warpage in thin-walled products without significantly increasing cycle time. Mohan et al. [6] provided a comprehensive review of the influence of process parameters on shrinkage and warpage, emphasizing the role of cooling system design. Nguyen et al. [7] used numerical simulations to study the impact of cooling channel design on warpage in thin-wall parts, finding that optimized layouts could significantly reduce deformation. Kuo and Xu [8] proposed a method for simultaneously improving warpage and cooling time, while Guerra et al. [9] highlighted the influence of both process parameters and post-molding conditions on the dimensional accuracy of complex parts. Nguyen et al. [10] investigated baffle cooling channels, showing their effectiveness in reducing displacement and improving roundness in circular parts. Earlier work by Ozcelik and Erzurumlu [11] compared warpage optimization methods, demonstrating the applicability of ANOVA, neural networks, and genetic algorithms. These studies collectively indicate that warpage can be mitigated through intelligent mold design and parameter optimization, often requiring a trade-off between cycle time and dimensional accuracy. Oktem et al. [12] applied the Taguchi optimization technique to minimize warpage and shrinkage variations in thin-shell plastic components. Using MoldFlow simulations based on L27 and L9 orthogonal arrays, the study employed signal-to-noise (S/N) analysis and ANOVA to identify optimal processing parameters. The optimized settings reduced warpage and shrinkage by 2.17% and 0.7%, respectively, confirming the effectiveness of the Taguchi method in improving dimensional stability of thin-walled injection-molded parts. Oktem [13] investigated the influence of key process parameters on volumetric shrinkage in ABS DVD-ROM covers produced by injection molding. Using Moldflow simulations based on a Taguchi L27 orthogonal design, a regression model was developed to describe the relationship between molding parameters and shrinkage behavior. ANOVA confirmed the model’s adequacy, and experimental validation showed strong agreement with simulation results. The study demonstrated that Taguchi-based modeling effectively predicts and minimizes shrinkage defects in plastic injection molding.

Conformal cooling channels—designed to follow the contours of the mold cavity—represent a significant advancement in thermal management for injection molding. Ahn and Park [14] pioneered laminated tooling with integrated conformal cooling channels, achieving more uniform temperature distribution compared to conventional straight-drilled channels. Pham et al. [15] introduced the feasibility of external gas-assisted mold-temperature control for thin-wall injection molding. Their study showed that this technique allows efficient regulation of mold surface temperature during the filling stage. As a result, it improved flow behavior, reduced residual stresses, and minimized warpage in thin-wall parts. The findings confirm that gas-assisted thermal control is an effective approach to enhance both dimensional accuracy and overall quality in thin-wall injection molding. Kariminejad et al. [16] demonstrated the advantages of sensorized metal AM mold inserts for real-time monitoring of cooling performance. Hassan and colleagues [17,18,19] conducted extensive research on cooling system modeling and parameter effects, establishing that optimized cooling not only improves thermal homogeneity but also reduces shrinkage and cycle time. Sánchez et al. [20] analyzed the relationship between cooling setup and warpage in injection molding. They showed that non-uniform cooling generates residual stresses and leads to part deformation. The study highlights that optimized cooling design is essential to reduce warpage and improve dimensional accuracy. Chung [21] developed an integrated method for designing conformal cooling channels and optimizing molding parameters for optical lenses. The results indicated that conformal cooling achieved better heat transfer and more uniform temperature distribution. This approach minimized defects and improved product quality in precision molding. More recent reviews, such as Kanbur et al. [22], have summarized state-of-the-art design and optimization strategies for conformal cooling channels, including their fabrication via metal additive manufacturing [23]. Applications of conformal cooling in high-performance tooling include the enhancement of cooling efficiency through hybrid fillers [24], rapid tooling integration [25], and improved tooling flexural strength [26]. Advanced designs, such as lattice structures [27], TPMS-based adaptive cooling [28], and baffle arrays [29,30], have further expanded the capabilities of conformal cooling in addressing localized thermal imbalances. Reggiani and Todaro [31] investigated a novel insert for extrusion dies produced by selective laser melting with conformal cooling channels. Their study showed that additive manufacturing enables complex channel geometries, which enhance cooling efficiency and improve thermal management. This approach demonstrates the potential of conformal channels in advanced tooling applications. Silva et al. [32] presented a review on the design, simulation, and optimization of conformal cooling channels in injection molds. They emphasized that conformal channels provide superior heat transfer compared to conventional designs. The review highlighted the role of simulation and optimization in achieving uniform temperature control and improving part quality. Studies by Torres-Alba et al. [33,34] introduced novel channel geometries to reduce residual stress and warpage, underscoring the continued evolution of cooling strategies. Overall, conformal cooling represents a transformative approach to mold temperature control, offering improvements in weld line strength, warpage reduction, and cycle time optimization.

While numerous studies have explored methods to improve weld line strength, reduce warpage, or enhance cooling efficiency individually, relatively few have integrated these objectives into a holistic optimization framework for injection-molded parts. In particular, the combined use of advanced cooling systems and statistical optimization methods—such as the Taguchi approach—offers a promising but underexplored pathway to achieving superior part quality. Recent studies on compliant mechanism design and optimization have highlighted the critical role of structural innovation and parameter refinement in improving accuracy and reliability of engineering systems. Dang et al. [35] introduced a one-degree-of-freedom compliant stage fabricated via additive manufacturing, demonstrating the potential of advanced fabrication methods for producing precise biomedical testing platforms. Building upon this foundation, a two-degree-of-freedom compliant stage was developed and optimized through a kinetostatic analysis-based approach combined with a neural network [36], underscoring the effectiveness of integrating analytical and intelligent computational tools for enhancing mechanical response. More recently, the same research group proposed a novel XYZ micropositioner embedded with a displacement sensor [37], achieving substantial improvements in precision and performance for biomaterial probing applications. Taken together, these works reflect a consistent research trajectory in which structural design, thermal or displacement control, and optimization via statistical or AI-based methods have proven essential for overcoming material-related limitations. This approach directly supports the present study, where mold-temperature regulation and parameter optimization are employed to enhance weld line strength in injection-molded plastic products

The present study addresses this gap by developing and evaluating a specialized injection mold equipped with a cooling layer designed to precisely regulate cavity temperature in the weld line region. While weld line behavior in plastic injection molding has been studied previously, novelty arises from combining targeted cavity temperature control with a Taguchi-based optimization approach. In particular, the integration of a mold with a dedicated cooling layer and the systematic evaluation of parameter effects under controlled conditions provide original contributions that distinguish this work from existing studies. Using the Taguchi method, three key process parameters—part thickness, melt temperature, and injection pressure—were systematically optimized to maximize weld line strength. The optimized parameters were then applied in actual production trials, yielding parts with markedly improved mechanical performance compared to those produced under standard conditions. By integrating targeted thermal control with robust statistical optimization, this research contributes to a deeper understanding of the interplay between process conditions and weld line properties. The findings are expected to guide both researchers and industrial practitioners in implementing cost-effective and efficient solutions for improving the quality of injection-molded plastic products, especially in applications where structural integrity at the weld line is critical.

## 2. Experimental

**Figure 1:**
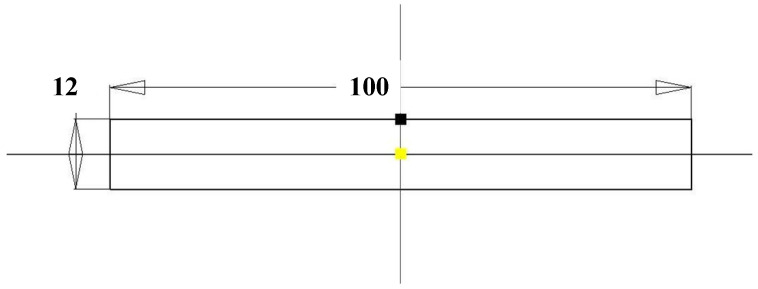
Specimen dimension for bending test (The yellow-colored square represents the center point of the molded product).

**Figure 2:**
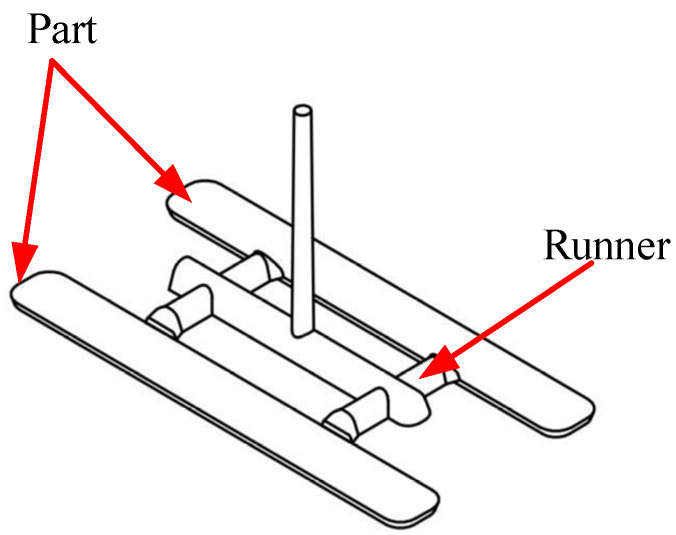
Three-dimensional design of plastic part for bending test.

Figure 1 illustrates the two-dimensional dimensions of the bending specimen. The bending test specimen was designed as a prismatic bar with an overall length of 100 mm and a rectangular cross-section. The specimen width was set to 12 mm, while the thickness was varied according to the experimental design. The geometry conforms to standard three-point bending test requirements, ensuring consistent stress distribution and minimizing geometric effects on the measured mechanical properties. The bending specimen mold was designed with two cavities, each part incorporating two gates (Figure 2). To ensure the consistent formation of weld lines, the two gates were positioned opposite each other so that the melt fronts met at the midline of the specimen. This design intentionally induces a weld line at the center region, allowing systematic evaluation of weld line strength under controlled processing conditions. The symmetric gate configuration also ensures balanced flow behavior, improving repeatability and reliability of the experimental results. The sprue serves as the connection between the machine’s nozzle and the runner system, functioning to deliver the heated molten plastic either into the runner channels or directly into the mold cavity (in runnerless mold configurations). The sprue bushing is the most commonly used sprue system due to its easy replacement and wide availability. A locating ring is attached to the front of the sprue bushing to ensure concentric alignment between the machine nozzle and the sprue bushing.

**Figure 3:**
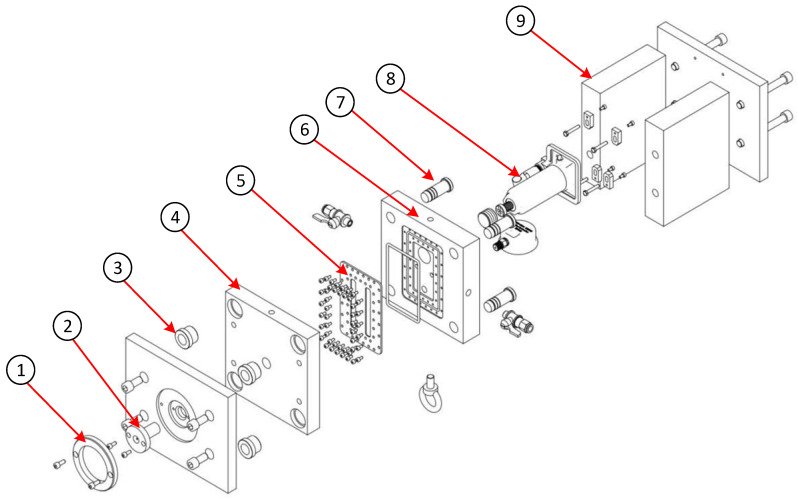
An exploded view of the injection mold assembly with cooling layer components. (1) Locating ring, (2) Sprue bushing, (3) Guide bushing, (4) Fixed clamping plate, (5) Insert plate with cooling channels, (6) Moving plate, (7) Guide pin, (8) Hydraulic cylinder, (9) Support plate.

A three-dimensional exploded view of the mold assembly incorporating detachable insert plates is shown in Figure 3. The mold assembly retains the fundamental components of a conventional two-plate mold, including the locating ring, sprue bushing, ejector pins, guide pins, guide bushes, return pins, core plate, and cavity plate. However, several modifications and enhancements were incorporated to accommodate research objectives. The key innovation lies in replacing the monolithic cavity block with a removable insert plate system. These inserts serve two primary functions: forming the product geometry during the injection molding cycle and transferring heat from the cavity to the external environment. For flexibility, the insert plates are secured to the fixed mold plate via hexagonal bolts, enabling quick removal and replacement when testing different specimen configurations. To ensure operational reliability, the system is sealed with rubber gaskets, preventing coolant leakage throughout the molding process. This design facilitates efficient experimentation with various product geometries and process parameters while maintaining the structural principles of a traditional two-plate mold.

**Figure 4:**
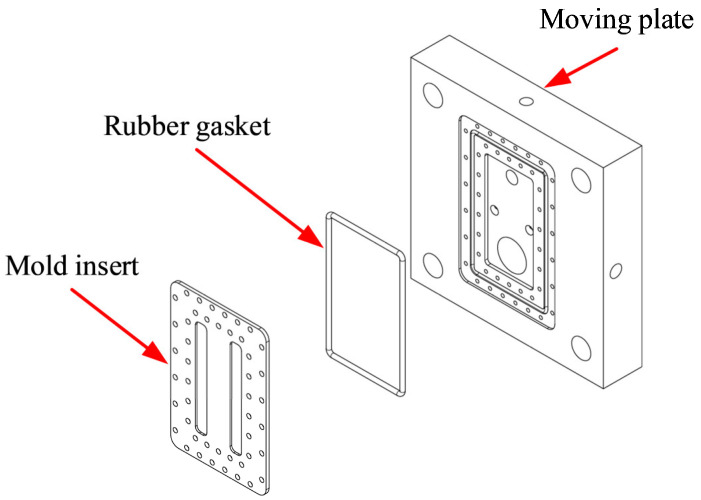
The mold cavity structure.

In injection molding, the concept of a thin thickness between the cooling channel and the cavity refers to a mold configuration in which the cooling channels are placed in close proximity to the cavity surface, thereby reducing the mold-wall thickness in this region. This design enables more efficient heat transfer between the coolant and the polymer melt, resulting in improved control of the cavity surface temperature during the molding cycle. Precise temperature control at the weld line enhances polymer chain interdiffusion and molecular bonding, thereby improving the flexural strength of molded parts. A thin mold-wall thickness between the cooling channel and the cavity accelerates thermal response and reduces mechanical weaknesses typically caused by weld lines. Figure 4 illustrates the structural configuration of the mold cavity consisting of the mold base, insert plate, and rubber gasket. The insert plate defines the cavity geometry, while the rubber gasket ensures proper sealing and prevents leakage during the injection molding process. The cavity wall thickness between the cooling channel and the mold cavity surface can be minimized by optimizing the insert design. Such a thin wall configuration improves thermal conductivity and enhances cooling efficiency, which directly contributes to better weld line quality and increased flexural strength of the molded parts. The introduction of a thin cooling layer as a means of mold temperature control constitutes a key novelty of this work. Unlike conventional cooling-channel arrangements, this design enables uniform and precise temperature regulation directly at the weld line region. This innovation not only enhances weld line flexural strength but also offers a novel approach to achieving accurate and stable mold temperature control in injection molding.

**Figure 5:**
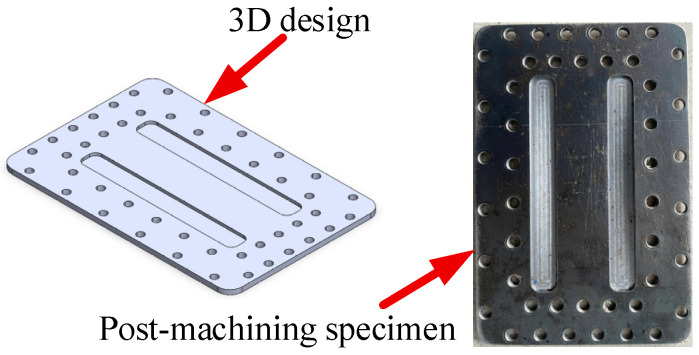
Design and machining of mold insert plate for bending test.

**Figure 6:**
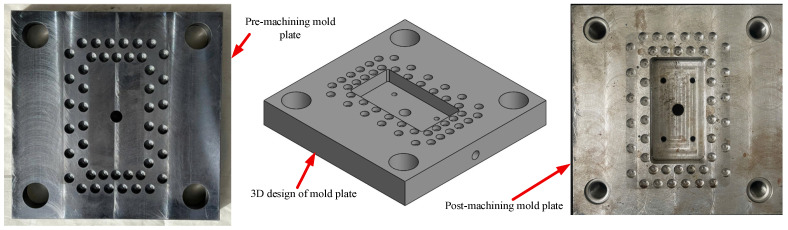
Design and machining of cavity mold plate.

**Figure 7:**
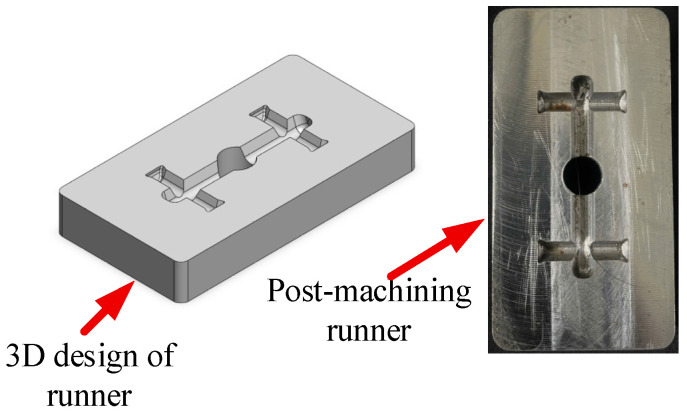
Design and machining of runner.

Figure 5, Figure 6 and Figure 7 illustrate the 3D designs alongside the corresponding post-machining components for three key parts of the injection mold: (Figure 5) the mold insert plate for bending test specimens, (Figure 6) the cavity mold plate, and (Figure 7) the runner. The mold insert plate is designed as a removable module, enabling high flexibility in molding various specimen geometries without requiring a complete mold reconfiguration. This modular approach facilitates rapid replacement and modification, making it especially suitable for experimental studies involving multiple part designs. The bending specimen was designed with rounded edges to prevent defects during the injection molding process. The mold cavity included a 5° draft angle to facilitate easy demolding. Its dimensions were adjusted to account for the shrinkage coefficient of the molded plastic specimen. In addition to forming the part geometry during injection, the mold insert integrates a cooling layer located beneath the cavity surface. This layer is engineered to regulate mold temperature effectively, thereby enhancing dimensional stability and surface quality while reducing cycle time. The cavity mold plate and runner were also designed with precision to ensure proper melt flow distribution. This design helps control weld line formation, aligning with the objectives of the bending strength optimization study.

**Figure 8:**
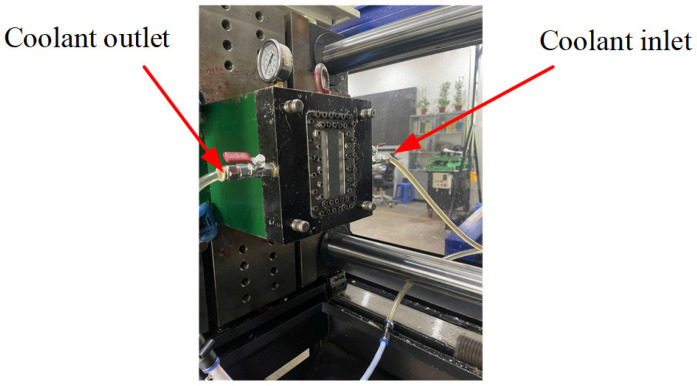
Cooling system for mold temperature control.

Figure 8 illustrates the mold temperature control process using a coolant circulation system. Coolant enters the mold through the inlet port and flows through the integrated cooling layer to adjust the cavity surface temperature as required. It is then discharged via the outlet port at the end of each injection molding cycle. This controlled thermal management ensures stable processing conditions, enhances dimensional accuracy, and improves the surface quality of the molded specimens

In all experiments, the material used was PA66 (Ultramid A3EG6, BASF, Ludwigshafen, Germany), a 30% glass-fiber-reinforced polyamide known for its high mechanical strength, dimensional stability, and thermal resistance. This engineering-grade polymer is widely utilized in structural and functional components across the automotive and electronics industries, where weld line strength and temperature resistance are critical to long-term performance. Its consistent processing behavior and relevance to industrial applications make it a suitable candidate for evaluating the effectiveness of cavity temperature control and process parameter optimization in improving weld line integrity.

**Figure 9:**
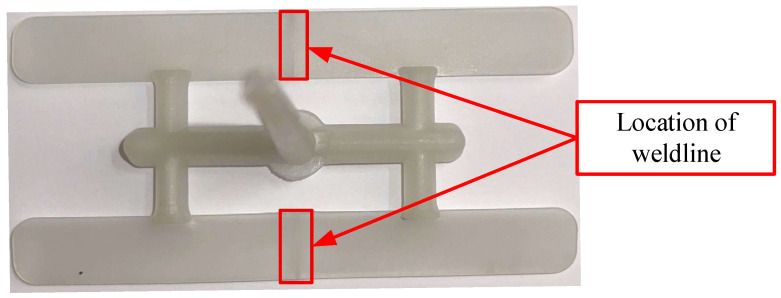
Weld line on actual bending test specimen.

Figure 9 presents the bending specimen produced in this experimental study. The injection-molded specimens were successfully filled to 100% without the occurrence of short shots, ensuring complete replication of the mold cavity. The weld line was observed at the midsection of the bending specimen. In general, weld lines form when two or more melt fronts converge during the filling stage, resulting in an interface with weaker molecular entanglement and reduced bonding strength. This structural discontinuity can act as a stress concentrator, reducing the molded product’s mechanical performance, especially its strength and durability under bending or impact loading.

**Figure 10:**
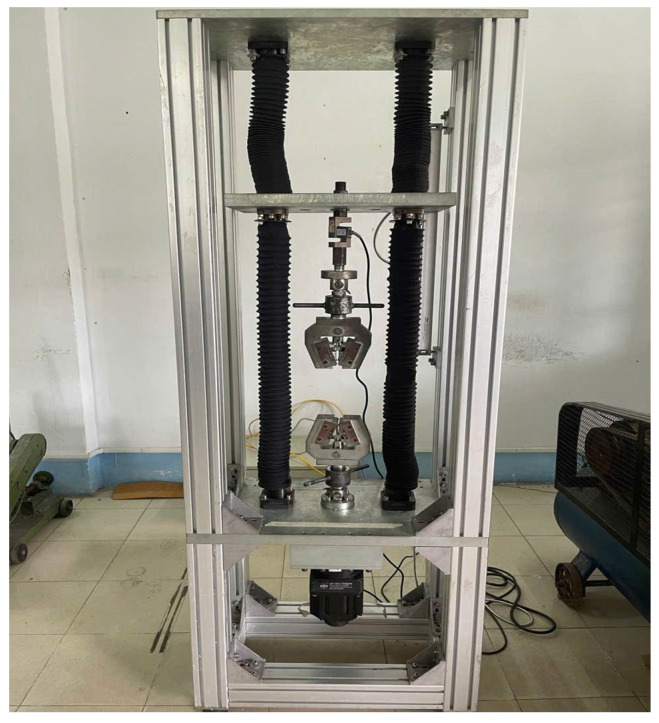
Flexural strength testing apparatus for specimen.

Figure 10 illustrates the experimental setup employed for evaluating the flexural strength of the molded specimens. The testing system consists of a rigid load frame equipped with upper and lower grips designed to secure the specimen during loading. The actuator, driven by an electric motor at the base of the frame, applies a controlled displacement or force in accordance with the programmed loading conditions. The apparatus enables three-point or four-point bending tests, ensuring accurate determination of flexural properties such as strength and modulus. The load cell and displacement transducer integrated into the system provide precise measurement of applied force and specimen deflection. This testing configuration is essential for characterizing the mechanical performance of injection-molded PA66 specimens. It is particularly useful for assessing the influence of weld line formation and processing parameters on flexural strength. The flexural strength of the molded specimens was measured following the ASTM D790 standard [38] for three-point bending tests. The tests were conducted at a crosshead speed of 2 mm/min, corresponding to a strain rate of approximately 0.01 s^−1^, with a span-to-depth ratio of 16:1 to ensure uniform stress distribution. For each experimental condition, ten specimens were tested, and the average value was reported as the flexural strength. All experiments were performed at room temperature (25 ± 2 °C) under controlled humidity.

## 3. Taguchi Method

The Taguchi method, originally developed by Genichi Taguchi, has been widely recognized as a robust statistical approach for process optimization and quality improvement. Its primary application lies in reducing variability in manufacturing systems while achieving higher performance at minimal cost. By employing orthogonal arrays, the method allows for efficient design of experiments, enabling researchers to systematically investigate the influence of multiple factors with a significantly reduced number of trials compared to full factorial designs. This efficiency makes the Taguchi method particularly suitable for industrial applications where experimental resources are limited. Compared with traditional experimental designs, the Taguchi approach not only reduces cost and effort but also enhances reproducibility and reliability of the results. These advantages have led to its extensive adoption across diverse fields, including polymer processing, where complex interactions among multiple parameters must be managed within practical experimental constraints.

One of the major advantages of the Taguchi method is its ability to provide clear insights into the relative significance of process parameters and their optimal settings, thereby guiding engineers toward improved product quality and reliability. Furthermore, the incorporation of the signal-to-noise (S/N) ratio ensures that the design is robust against noise factors, enhancing consistency in real production environments. However, the method also presents certain limitations, including difficulties in handling complex interactions among parameters and its reliance on simplified assumptions regarding linearity of effects. Despite these drawbacks, the Taguchi method remains an effective tool in experimental design, offering a practical balance between accuracy, resource efficiency, and applicability across diverse engineering problems. The signal-to-noise (S/N) ratio, proposed by Taguchi, is a key performance index used to measure the robustness of a system by quantifying the variation in quality characteristics relative to their desired values. It represents the relationship between the mean (signal) and the standard deviation (noise) of a response variable. This allows simultaneous optimization of both the average performance and its variability. The goal is to maximize the S/N ratio so that the product or process is less sensitive to uncontrollable factors (noise). In this experiment, the S/N ratio was calculated using the larger-the-better criterion.

(1) $$S/N=-10{log}_{10}\left[\frac{1}{n}\sum_{i=1}^{n}\frac{1}{y_{i}^{2}}\right]$$

Here, y_i_ represents the measured experimental values, and n refers to the number of specimens in each trial.

The experimental design was based on the Taguchi method with three control factors, each varied at five levels (Table 1). To balance experimental efficiency with statistical robustness, an L25 orthogonal array was employed, requiring only 25 trials instead of 125 for a full factorial (53) design (Table 2). Each factor was assigned to one column of the orthogonal array, ensuring orthogonality and independence of the main effects. The part thickness of the molded specimen was varied across five levels (0.75, 1.00, 1.25, 1.50, and 1.75 mm), representing thin-to-thick configurations to investigate the influence of material volume on weld line strength. The filling pressure was controlled at five levels (12, 13, 14, 15, and 16 MPa), covering a practical molding range in which higher pressures improve cavity filling but may also introduce residual stresses. Similarly, the melt temperature was adjusted to 250, 255, 260, 265, and 270 °C, capturing the effect of polymer viscosity and molecular interdiffusion on weld line formation and strength. This design provides balanced coverage of the entire parameter space. For example, Experiment No. 1 corresponds to the lowest thickness (0.75 mm), medium pressure (13 MPa), and the lowest melt temperature (250 °C), whereas Experiment No. 25 combines the highest levels of all factors (1.75 mm thickness, 16 MPa pressure, 270 °C melt temperature). Such a distribution enables systematic identification of main effects and interactions influencing the weld line flexural strength of the molded specimens. It should be noted that holding pressure and cooling rate were not selected as control factors in this study. For thin-walled parts, the molten polymer solidifies rapidly after the filling stage, minimizing the influence of the holding phase and cooling variation. Therefore, these parameters were considered secondary and excluded from the optimization design to focus on the most dominant factors affecting weld line strength. The flexural strength at the weld line was the main performance characteristic. The signal-to-noise (S/N) ratio was calculated using the larger-the-better criterion, emphasizing maximization of weld line strength. In the present work, three parameters were prioritized as the primary control factors; however, it is recognized that additional variables—such as screw rotation speed, back pressure, holding pressure, mold temperature, packing pressure, and packing time—may also exert significant influence on weld line strength. To preserve the feasibility and focus of the experimental design, these parameters were not included in the L25 orthogonal array. Their potential effects merit systematic investigation in future studies to provide a more comprehensive understanding of weld line behavior.

## 4. Results and Discussion

### 4.1. Mold Temperature Control Based on the Thickness of the Layer Between the Cooling Channel and Cavity

**Figure 11:**
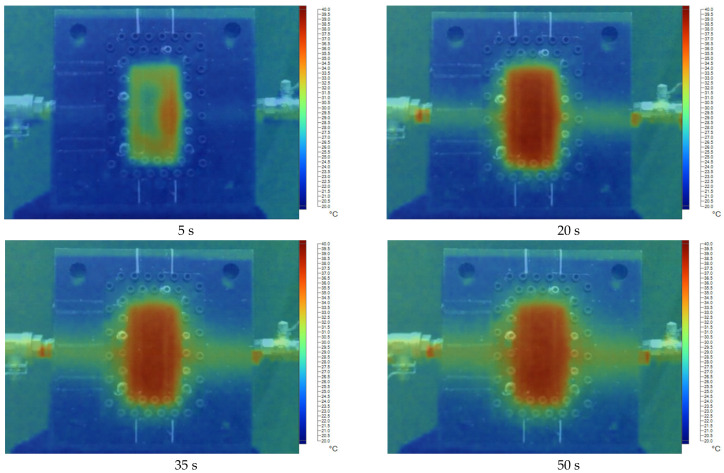
The temperature distribution results at 40 °C for the flexural specimen with a thickness of 1 mm at 5 s, 20 s, 35 s, and 50 s.

**Figure 12:**
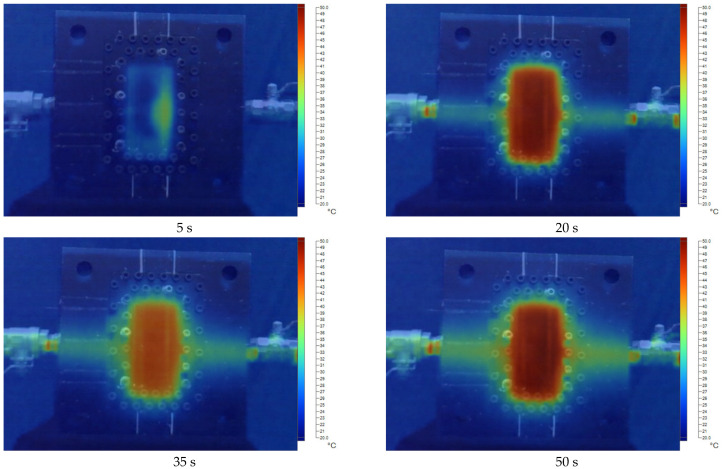
The temperature distribution results at 50 °C for the flexural specimen with a thickness of 1 mm at 5 s, 20 s, 35 s, and 50 s.

**Figure 13:**
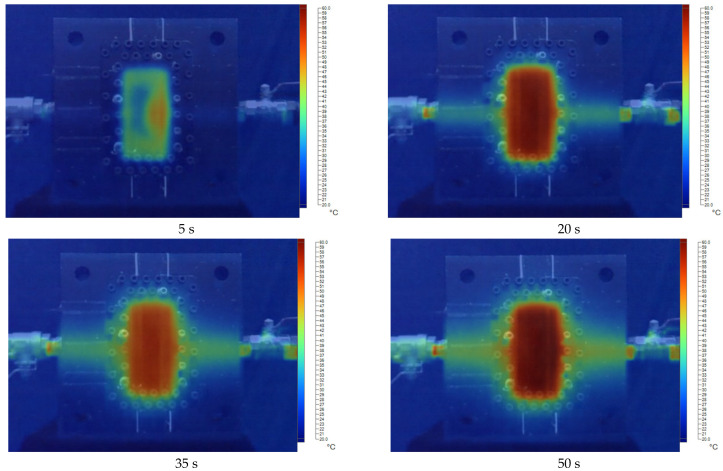
The temperature distribution results at 60 °C for the flexural specimen with a thickness of 1 mm at 5 s, 20 s, 35 s, and 50 s.

**Figure 14:**
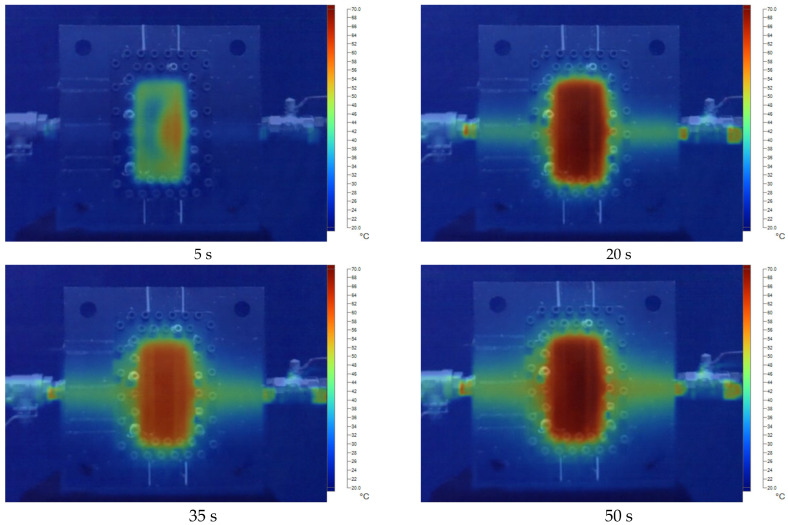
The temperature distribution results at 70 °C for the flexural specimen with a thickness of 1 mm at 5 s, 20 s, 35 s, and 50 s.

**Figure 15:**
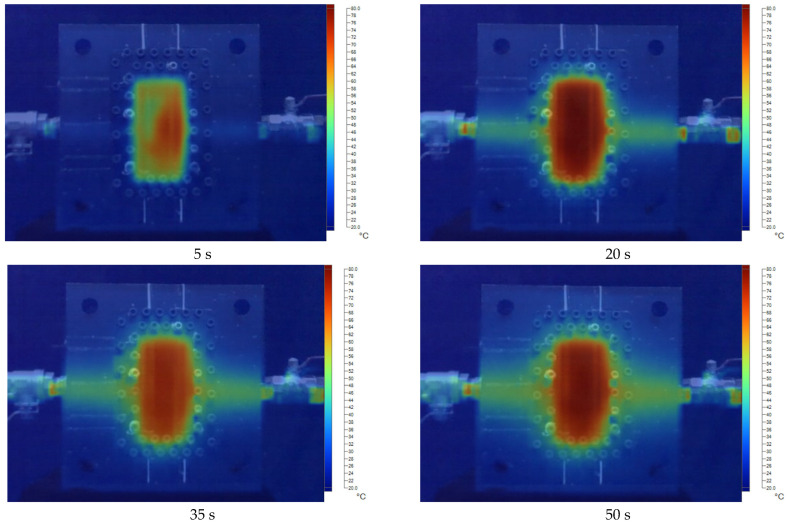
The temperature distribution results at 80 °C for the flexural specimen with a thickness of 1 mm at 5 s, 20 s, 35 s, and 50 s.

Figure 11, Figure 12, Figure 13, Figure 14 and Figure 15 present the thermal distribution results for flexural specimens with a thickness of 1 mm at mold temperatures ranging from 40 °C to 80 °C, observed at different time intervals (5 s, 20 s, 35 s, and 50 s). At 40 °C (Figure 11), the heating process progresses slowly, and even after 50 s, a significant thermal gradient remains between the specimen center and its edges. This indicates limited heat transfer efficiency at relatively low mold temperatures. At 50 °C (Figure 12), heat conduction accelerates, and the temperature distribution becomes more uniform after 35–50 s. Nevertheless, a certain degree of non-uniformity persists, particularly at the early stage (5 s). At 60 °C (Figure 13), the heating process is noticeably enhanced. By 20 s, the specimen core already reaches a stable hot region, while the periphery shows a faster response compared to lower mold temperatures. At 70 °C (Figure 14), uniformity improves further, and the temperature field achieves near-homogeneous distribution by 35 s. The central hot zone stabilizes more quickly, reducing the thermal gradient across the specimen. At 80 °C (Figure 15), the thermal distribution becomes the most uniform among all investigated conditions. By 20–35 s, the entire specimen reaches a steady-state thermal condition, minimizing temperature gradients and ensuring consistent heat transfer throughout the surface and core. The results clearly demonstrate that increasing mold temperature enhances the uniformity of thermal distribution across the flexural specimens. As the mold temperature rises from 40 °C to 80 °C, the heating process becomes progressively faster, and the temperature field stabilizes earlier, with markedly reduced gradients. Importantly, the thin-walled mold configuration plays a decisive role in this process. The reduced wall thickness promotes efficient heat conduction between the cooling channels and the mold cavity, thereby enabling better temperature regulation and faster homogenization of the thermal field. Mechanistically, improved thermal uniformity promotes a more consistent solidification rate across the molded part, thereby reducing local variations in shrinkage between hot and cold regions. This uniform cooling behavior effectively minimizes the development of residual stresses, leading to enhanced dimensional stability and improved mechanical integrity—particularly in regions near the weld line.

**Figure 16:**
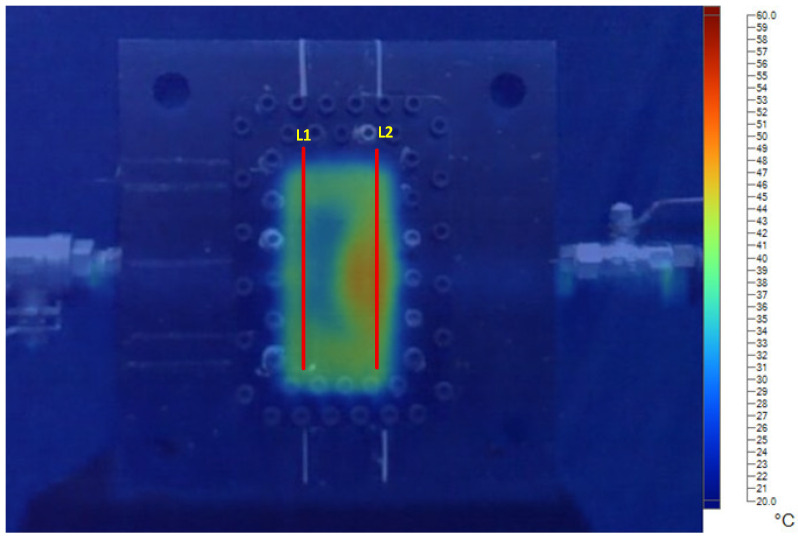
Temperature measurement lines (L1 and L2) along the mold cavity.

Figure 16 illustrates the locations of two measurement lines, L1 and L2, which correspond to the temperature monitoring positions of the bending test specimens. L2 is located near the inlet of the heating water, whereas L1 is positioned near the outlet.

**Figure 17:**
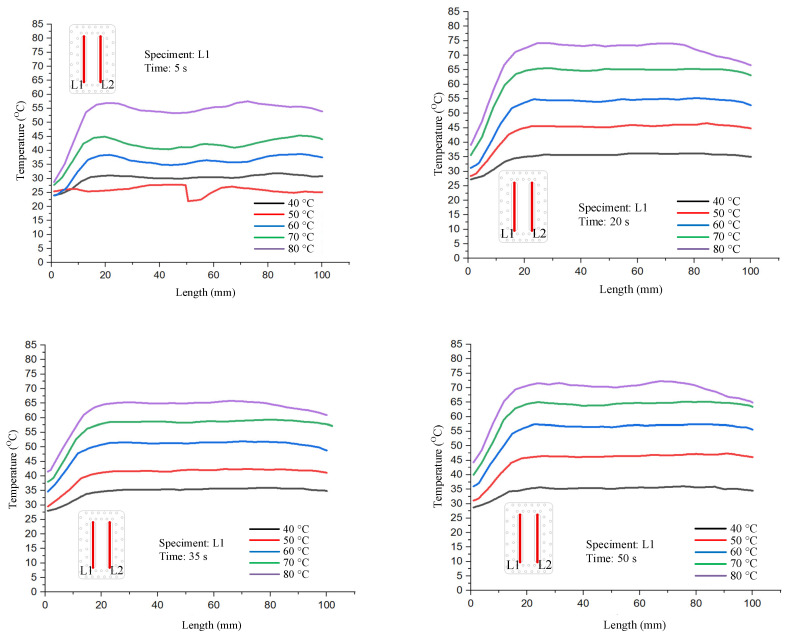
Temperature distribution along line L1 under different mold temperature conditions with a product thickness of 1 mm.

**Figure 18:**
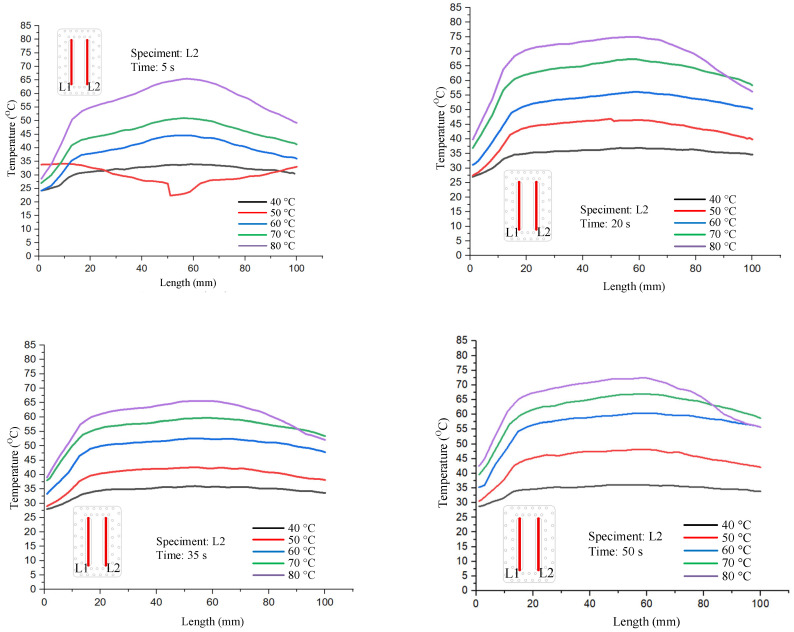
Temperature distribution along line L2 under different mold temperature conditions with a product thickness of 1 mm.

Figure 17 and Figure 18 show the temperature distribution along line L1 and L2 under different mold temperature conditions with a product thickness of 1 mm. At early stages, the distribution is less stable, while more uniform profiles are observed at later times as the thermal field stabilizes. At the initial stages of heating, the mold temperature is not yet stabilized, resulting in more scattered and less uniform distributions. As time progresses, the temperature profiles along both L1 and L2 become smoother and more uniform. This indicates a gradual stabilization of the thermal field within the mold cavity. A consistent trend is observed: the temperature along line L2, located near the heating-water inlet, is always higher than that along line L1, which is closer to the outlet. This difference persists regardless of the set mold temperature. This behavior highlights the influence of the inlet–outlet positioning on thermal uniformity. Notably, at higher mold temperatures (70–80 °C), the temperature difference between L1 and L2 becomes more pronounced. This is attributed to the combined effects of enhanced heat transfer at the inlet region and greater heat loss at the outlet. Together, these factors amplify the thermal gradient across the cavity.

### 4.2. Enhanced Flexural Strength Through Thin Cooling Layer Between the Channel and Cavity for Weld Line Improvement in Injection-Molded Plastics

**Figure 19:**
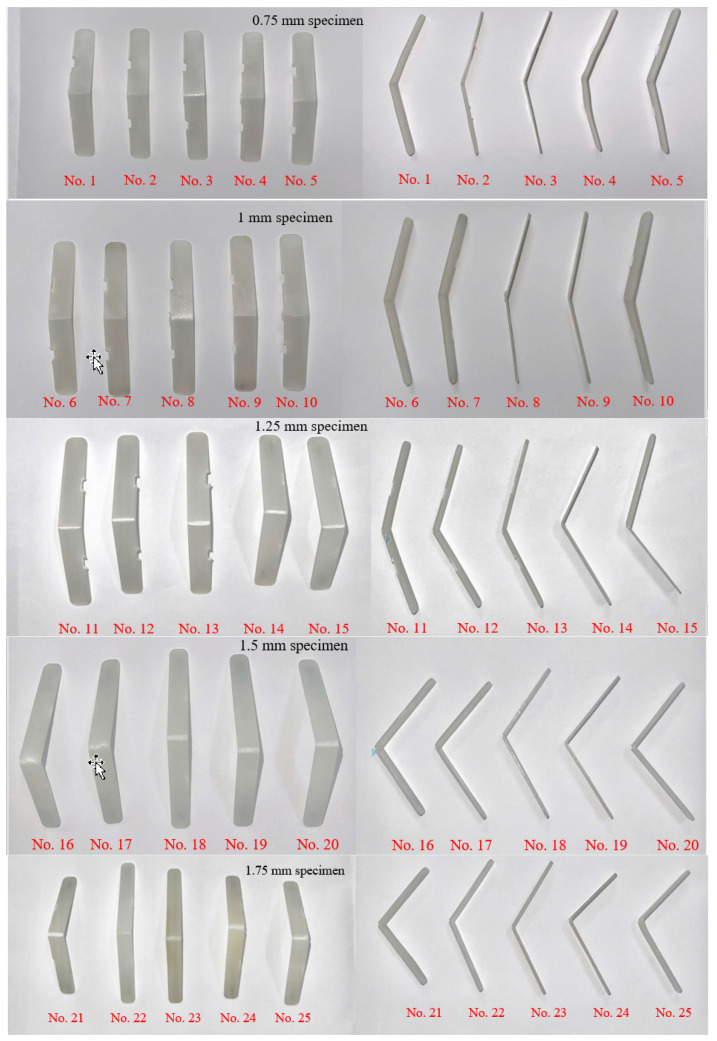
The actual product after bending with a product thickness of 0.75 mm, 1 mm, 1.25 mm, 1.5 mm, and 1.75 mm.

**Figure 20:**
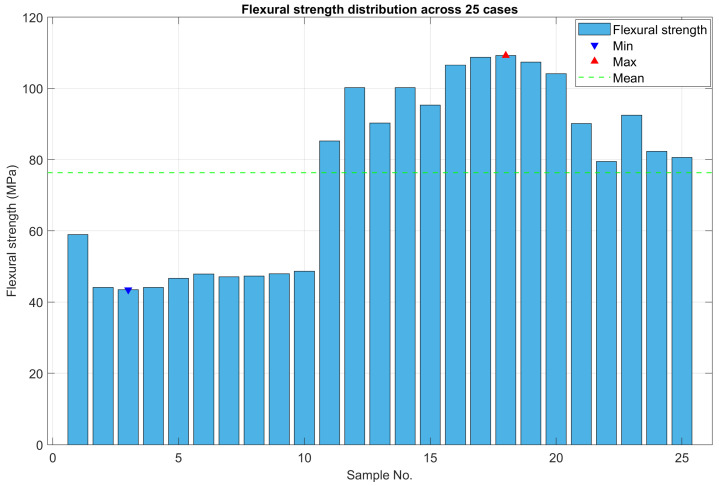
Statistical chart of maximum stress for 25 experimental cases.

The molding results of flexural specimens with different thicknesses of 0.75 mm, 1 mm, 1.25 mm, 1.5 mm, and 1.75 mm are presented in Figure 19. The statistical distribution of maximum flexural stress for 25 experimental cases is illustrated in Figure 20. All tested bending specimens experienced failure within the strain range of 5% to 10%, which is consistent with typical brittle–ductile transition behavior observed in polymeric materials. The highest recorded maximum stress among the 25 cases was 109.23 MPa, whereas the lowest was 43.42 MPa. Overall, the flexural strength values obtained from the experimental series ranged from 43.42 MPa to 109.23 MPa. When compared to the manufacturer’s reference flexural strength values of 220–250 MPa, it is evident that the experimental results show significantly lower strength. This discrepancy can be attributed primarily to two factors. First, the testing conditions applied in this study may differ from those specified by the manufacturer, including strain rate, temperature, or specimen geometry. Second, the specimen fabrication process employed by the research group may have introduced defects, microvoids, or weld lines, which are known to reduce the effective flexural strength of polymeric samples. These findings highlight the sensitivity of flexural strength to processing and testing conditions and emphasize the need for strict control of sample preparation and experimental procedures to obtain results comparable to manufacturer specifications.

Table 3 presents the ANOVA results for flexural strength (MPa), showing the statistical contribution of each process parameter—part thickness, filling pressure, and melt temperature—to the weld line strength. The ANOVA results demonstrate that part thickness had a statistically significant effect on the flexural strength of the weld line specimens (F = 128.36, *p* < 0.001), indicating a very strong dependence of weld line strength on the geometric dimension of the molded part. The *p*-value well below 0.05 confirms that the observed differences among thickness levels are unlikely to be due to random variation. In contrast, filling pressure (*p* = 0.401) and melt temperature (*p* = 0.953) did not show statistically significant effects (*p* > 0.05), suggesting that within the experimental range, their influence on flexural strength is relatively weak or masked by the dominant contribution of thickness. From a statistical perspective, the low *p*-value for part thickness implies a very low probability that the observed effect occurred by chance. This validates that part thickness is the most influential factor affecting weld line integrity, while the other parameters contribute only marginally to the overall variation in mechanical performance. It is acknowledged that the present Taguchi analysis was limited to three primary process parameters—part thickness, filling pressure, and melt temperature. While this approach effectively demonstrated the influence hierarchy and verified the impact of cavity temperature regulation on weld line strength, the number of factors was insufficient to establish a reliable regression or interaction model. Future studies may extend the analysis to include additional parameters, such as holding pressure and cooling rate, to develop a more comprehensive predictive framework.

**Figure 21:**
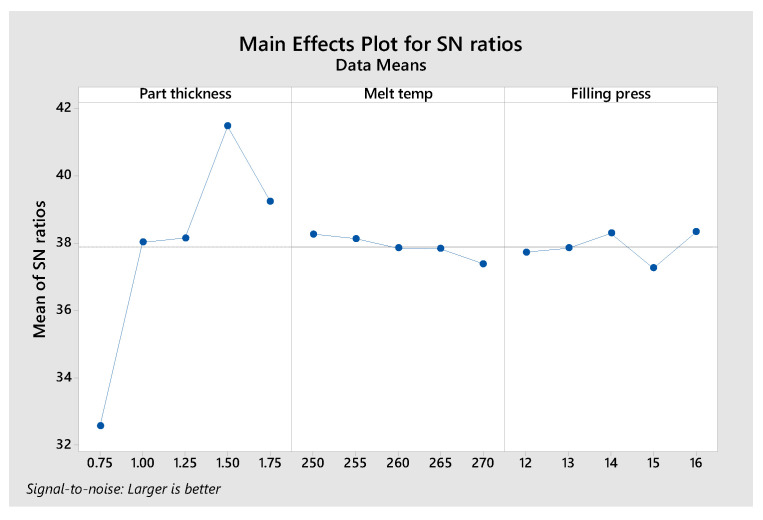
Analysis of the influence of factor levels on maximum stress.

The main effects plot for the SN ratios (Figure 21) provides insights into the relative influence of part thickness, melt temperature, and filling pressure on maximum flexural stress. For part thickness, the SN ratio shows a substantial upward trend from 0.75 mm to 1.5 mm, where it reaches its peak value. Beyond 1.5 mm, the ratio slightly declines, suggesting diminishing benefits of further thickness increase. This highlights that 1.5 mm is the optimal thickness for maximizing stress resistance. Regarding melt temperature, the SN ratios remain relatively stable across the investigated range of 250 °C to 270 °C, showing minimal fluctuations. This indicates that melt temperature exerts only a marginal effect on maximum stress compared with the other factors. For filling pressure, the SN ratio increases with pressure and attains its highest value at 16 MPa. This outcome implies that higher injection pressures enhance maximum stress by improving compaction and reducing void formation in the molded parts. Overall, the analysis clearly identifies part thickness as the most influential factor, followed by filling pressure, while melt temperature exerts the least significant impact under the tested conditions. This ranking underscores the dominant role of structural dimensions (thickness) in governing the mechanical performance of molded parts. It is acknowledged that the Taguchi L25 orthogonal array primarily emphasizes main effects; consequently, the analysis of interaction effects in this study is limited. The design was intended to identify the dominant factors governing weld line strength within a feasible experimental scope, though this approach may inherently underestimate the coupling influences among parameters. Nevertheless, based on the experimental observations, some preliminary hypotheses can be proposed regarding possible interactions. When the part thickness decreases, the flow resistance inside the cavity increases, leading to slower melt flow and faster cooling, which adversely affects weld line quality. Melt temperature also influences the filling behavior by altering the viscosity of the polymer melt. Overall, these three parameters are likely to interact with each other, and their coupled effects will be further investigated in future studies.

**Figure 22:**
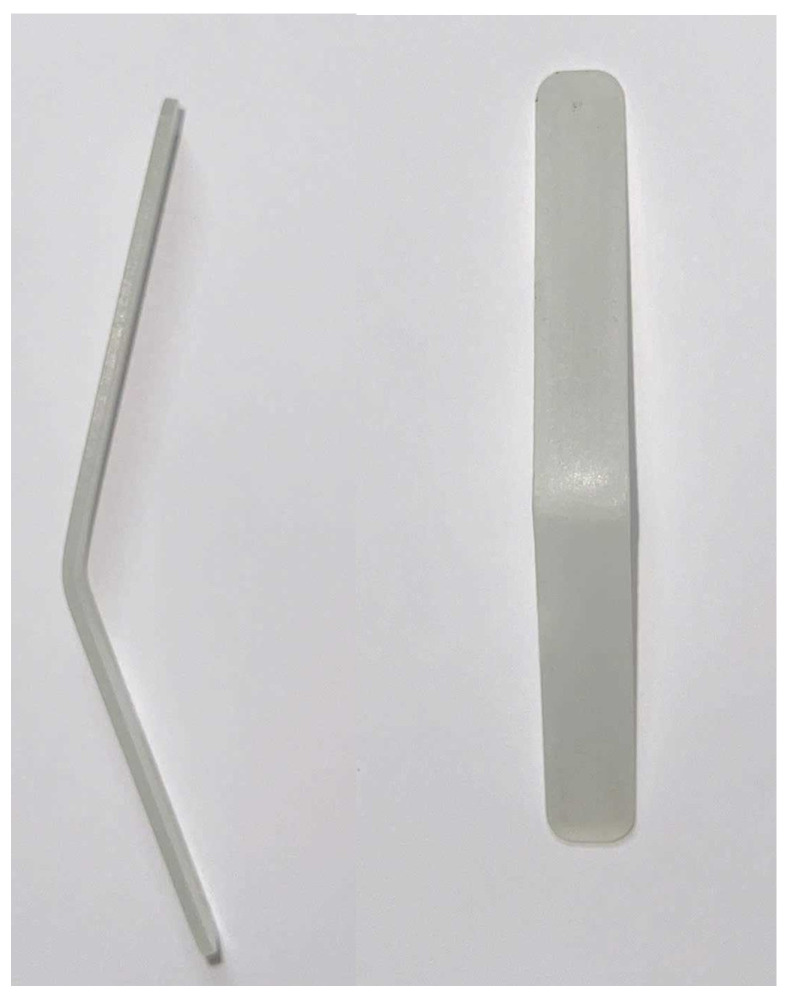
Actual specimen with optimal parameter set.

Using the Taguchi optimization approach, the optimal parameter set was identified as a part thickness of 1.5 mm, a melt temperature of 250 °C, and a filling pressure of 16 MPa, as shown in Table 4. Based on this configuration, an experimental molding and bending test was conducted. The actual molded specimen produced under these optimal conditions is presented in Figure 22, demonstrating the effectiveness of the Taguchi method in determining suitable process parameters for enhancing specimen quality.

**Figure 23:**
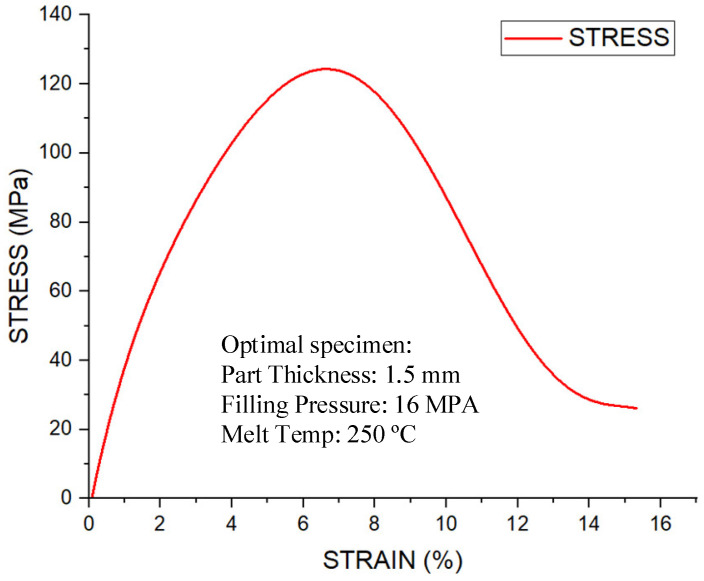
Stress–strain diagram of optimal specimen.

A stress–strain diagram in the context of a flexural (bending) test illustrates the relationship between flexural stress (the bending stress at the specimen’s outer surface) and flexural strain (the corresponding deformation relative to the original geometry) under bending loads. Figure 23 shows the stress–strain curve of the optimal specimen. Under the optimal parameter set—a melt temperature of 250 °C, a part thickness of 1.5 mm, and a filling pressure of 16 MPa—the stress–strain response shows a pronounced linear-elastic region from the origin to the proportional limit. Beyond this point, a gradual non-linear segment appears as micro-damage localizes near the weld line. The curve reaches an ultimate flexural stress on the order of the best measured value for this study (≈121.88 MPa at the weld line section), after which softening precedes final fracture. Consistent with the overall test population, failure occurs within a moderate deformation window (≈5–10% strain), indicating adequate ductility while maintaining high peak load capacity. In general, the stress–strain diagram corresponds to a flexural (bending) test, exhibiting a linear elastic region, yielding behavior, peak flexural strength, and subsequent failure. The stress–strain diagram of the optimally molded specimen confirms high stiffness and strength, along with stable post-yield behavior. This combination results in the maximum weld line flexural stress observed in the study. This validates the strategy of combining cavity-temperature management (via a thin cooling layer) with Taguchi-guided parameter selection to upgrade weld line integrity and overall mechanical performance in injection-molded parts.

## 5. Conclusions

This study has demonstrated that weld line strength in injection-molded plastic parts can be effectively enhanced by integrating cavity temperature control with statistical optimization of process parameters. The main conclusions are summarized as follows:

o Localized temperature control: The use of a cooling-layer-equipped mold enabled localized thermal regulation, resulting in improved weld line strength.
o Systematic optimization: The Taguchi method facilitated an efficient evaluation of the effects of part thickness, melt temperature, and injection pressure on weld line strength.
o Experimental results: The weld line flexural strength ranged from 43.426 MPa to 109.235 MPa, with the maximum value of 121.880 MPa obtained under optimal conditions—a 250 °C melt temperature, a 1.5 mm part thickness, and a 16 MPa injection pressure.
o Factor influence: Part thickness was found to be the most dominant factor affecting weld line strength, followed by injection pressure, while melt temperature had only a marginal impact within the tested range.

The findings highlight the essential role of thermal management during molding and demonstrate that the proposed Taguchi-based optimization provides a practical and reliable strategy for establishing robust processing conditions and enhancing the structural integrity and service performance of injection-molded plastic products, particularly in applications requiring high durability.

## Figures and Tables

**Table 1:** The input factors and levels.

No.	Factor	Level 1	Level 2	Level 3	Level 4	Level 5
1	Part Thickness (mm)	0.75	1	1.25	1.5	1.75
2	Filling Pressure (MPa)	12	13	14	15	16
3	Melt Temperature (°C)	250	255	260	265	270

**Table 2:** Taguchi orthogonal array L25 (5^6^) corresponding to three input factors, each at five levels.

No.	Part Thickness (mm)	Filling Pressure (MPa)	Melt Temperature (°C)
1	0.75	13	250
2	0.75	14	255
3	0.75	15	260
4	0.75	16	265
5	0.75	12	270
6	1	14	250
7	1	15	255
8	1	16	260
9	1	12	265
10	1	13	270
11	1.25	15	250
12	1.25	16	255
13	1.25	12	260
14	1.25	13	265
15	1.25	14	270
16	1.5	16	250
17	1.5	12	255
18	1.5	13	260
19	1.5	14	265
20	1.5	15	270
21	1.75	12	250
22	1.75	13	255
23	1.75	14	260
24	1.75	15	265
25	1.75	16	270

**Table 3:** ANOVA Table for Flexural Strength (MPa).

No.	Source	df	Sum of Squares	F-Value	*p*-Value
1	Part Thickness (mm)	4	15,002.66	128.36	<0.001
2	Filling Pressure (Mpa)	4	128.56	1.10	0.401
3	Melt Temperature (°C)	4	19.07	0.16	0.953
4	Residual (Error)	12	350.64	–	–

**Table 4:** The optimal parameter set.

Figure 1	Value
Part Thickness (mm)	1.5
Filling Pressure (MPa)	16
Melt Temperature (°C)	250

## Data Availability

The data used to support the findings of this study are available from the corresponding author upon request.
